# Evaluation of collagen sponge and xenografts versus collagen sponge alone in alveolar ridge preservation: a randomized controlled clinical trial

**DOI:** 10.1038/s41598-025-28579-1

**Published:** 2025-12-06

**Authors:** Hend Magdy Mohamed Mahmoud, Hala Helmi Hazzaa, Zienab Shalaby Farid, Naglaa El-Wakeel

**Affiliations:** 1 Periodontist at 6 of October Military Forces Hospital, Ministry of Defence, Cairo, Egypt; 2 Oral Medicine, Periodontology, Diagnosis and Radiology Department, Faculty of Dental Medicine for Girls, Al-Azhar University, Cairo, Egypt

**Keywords:** BARP technique, Deproteinized bovine bone mineral, CBCT superimposition, Histomorphometry, Keratinized tissue, Biological techniques, Medical research

## Abstract

This study evaluated the clinical, radiographic, and histological outcomes of alveolar ridge preservation (ARP) using collagen sponge with xenografts (CS + Xenografts) versus collagen sponge (CS) alone, compared to spontaneous healing. 36 extraction sockets were randomly allocated into three groups: Group I (CS + Xenografts), Group II (CS alone), and Group III (control) spontaneous healing. Soft tissue assessment and CBCT imaging were conducted before tooth extraction (baseline) and 6 months post-extraction, followed by histologic and histomorphometric analysis of bone biopsies. Groups I and II exhibited minimal vertical and horizontal soft tissue changes compared to the control group (*P* < 0.001), with no statistically significant difference between Group I and II (*P* ≥ 0.05). Vertical and horizontal bone resorption was significantly lower in Groups I and II than in the control group (*P* < 0.001), with no statistically significant difference between Groups I and II regarding vertical bone loss (*P* = 0.477 and 0.108, respectively); percent of changes were 8.68 ± 2.69 and 8.61 ± 2.14 respectively. The greatest reduction in alveolar bone width was observed at 1 mm: 17.81 ± 3.97 (Group I), 19 ± 2.77 (Group II), and 41.79 ± 10.3 (Group III); overall *P* < 0.001. Histologically, Group I had the highest area% of lamellar bone and no residual inflammation, followed by Group II, which showed more inflammation; Group III had the lowest area% of lamellar bone. Intervention techniques were clinically and radiographically proven effective in ARP, however, CS + Xenografts histological results showed more lamellar bone and less residual inflammations.

## Introduction

Tooth extraction causes disuse atrophy of the surrounding soft tissues and alveolar bone. Within 1 year of extraction, an average of 50% of the ridge width is reduced with an average amount of loss between 5 and 7 mm, and 2/3 of this reduction occurs within the first 3 months, being more prominent on the buccal side compared to the lingual/palatal side^1^, Further, an average 4.0–4.5 mm of horizontal bone resorption following atraumatic extraction has been reported^2^. These post extraction changes deeply alter the proper environment for both dental prosthesis and implant placement. Thus, performing reformative technologies at fresh extraction socket is important to reduce the need for further tissue augmentations essential for aesthetics and implantation at this site^3^.

Alveolar ridge preservation (ARP) is a predictable way to reduce undesirable horizontal and vertical ridge reduction following extraction when dental implant treatment is to be delayed^4^. Several approaches have been utilized for ARP, most of which consist of the placement of bone grafts, soft tissue grafts, use of membranes, growth factors, or a combination of all to reduce alveolar loss in height and width^5^. With the available literature, different mechanisms are introduced to achieve a good ARP and many controversies revolve around such a broad topic^6^.

Xenograft is a commonly used bone graft in dental field, being physically and chemically identical to natural human bone mineral plus being highly osteoconductive material^7^. ARP with xenografts was shown to be effective in reducing post-extraction ridge changes in the aesthetic region^8^. Collagen is a familiar material in dental clinics for decades, being highly porous, biocompatible, and biodegradable, thus, collagen was proposed as potential alternative material for ARP combined with bone grafts and have been used as socket seal material^9^. collagen sponge were used to fill extraction socket aiming to stabilize clot and granulation tissue formation and to reduce postoperative pain^10^, besides offering a favorable environment for the osteoblast’s attachment and proliferation. However, using collagen sponge alone in ARP is still controversial in clinical practice and more evidences are needed to support its use in ARP^11^.

In 2021, a new technique, the Biologically-oriented Alveolar Ridge Preservation (BARP) for ARP was introduced, it restricts socket grafting to the coronal portion of the socket^12^. A 2019 meta-analysis didn’t identify an ideal approach for alveolar ridge preservation with superior outcomes^13^. In the field of regenerative dentistry, advancements in techniques and biomaterials, have shown promising potential in restoring damaged tissues, serving as a temporary framework for cells to adhere, proliferate, and differentiate into functional tissues^14^. Limited data are available through randomized controlled trails with multiple evaluation methods on the BARP technique for socket preservation. Further as this technique partially utilize the collagen sponge to fill the extraction socket, we thought to test the efficacy of BARP versus collagen sponge in socket preservation. Thus, the aim of the present study was to evaluate the effect of BARP versus collagen sponge in socket preservation clinically, radiographically and histologically and all compared to spontaneous healing socket as control.

## Materials and methods

### Study design and population

This double blinded randomized controlled clinical trial was carried out on 36 healthy individuals aged between 20 and 60 years, who needed implant treatment after anterior/premolar tooth extraction. Subjects were recruited from outpatient clinic of the Department of Oral medicine and Periodontology, Faculty of Dentistry for Girls, Al-Azhar University, Cairo, Egypt. The study was registered on clinical trial .gov (NCT06896097) (https://register.clinicaltrials.gov/prs/beta/records), was conducted following the Helsinki Declaration, and the protocol was approved by the Clinical Researches Ethics Committee of Faculty of Dentistry for Girls, Al-Azhar University (REC-ME-25-03).

**Fig. 1:**
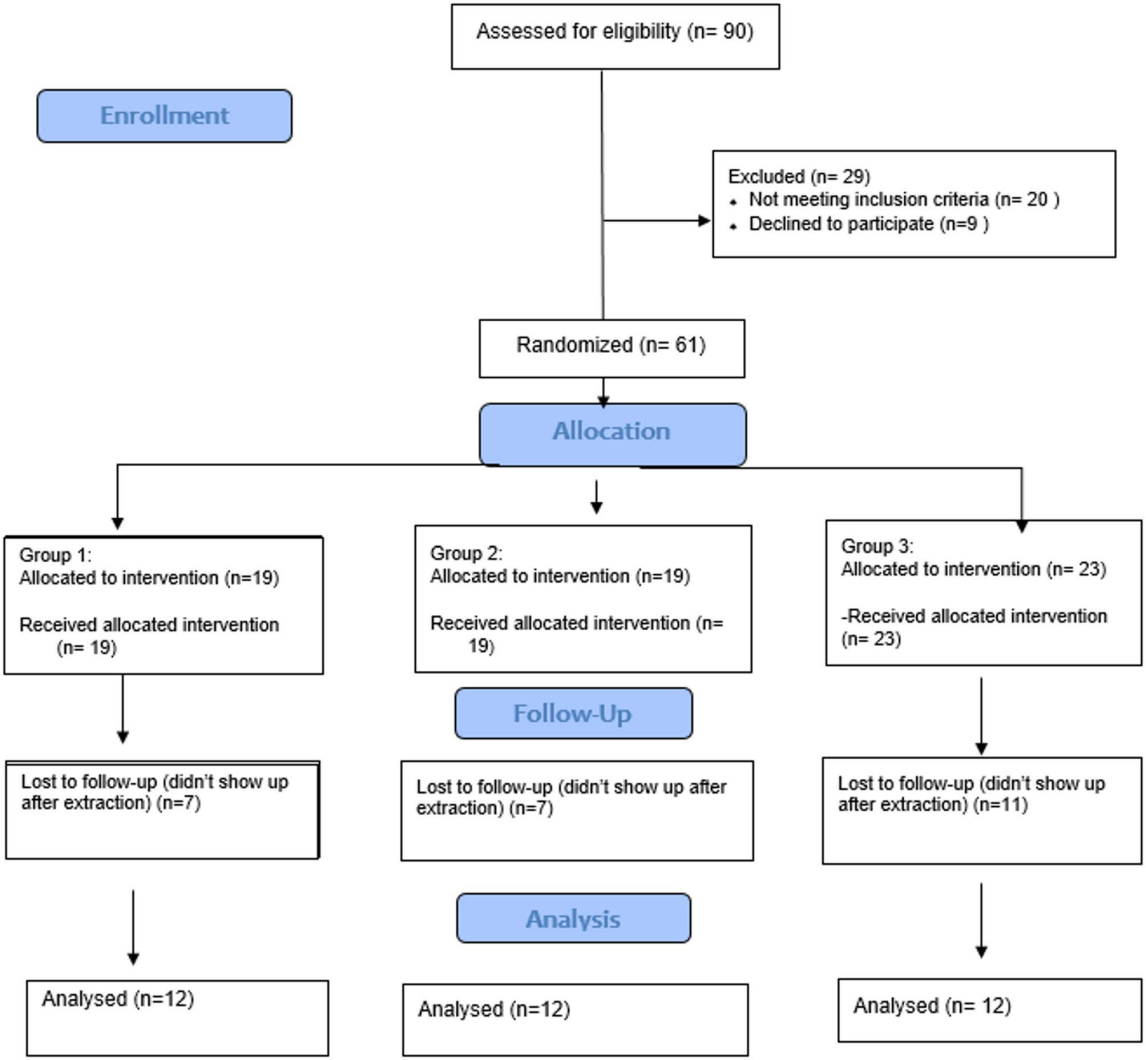
The CONSORT flowchart for this randomized controlled clinical trial.

Subjects were randomly assigned into one of three groups: group I: ARP using collagen sponge + xenograft, group II: ARP using collagen sponge, and group III: negative control post extraction socket. Subjects randomization was done using software program (www.randomization.com). Subjects were blinded to group allocation; surgical treatments were performed by one investigator (H.M). All study outcomes were assessed by one blinded examiner per each outcome (Z.S. for clinical outcome, E.H. for Radiographic outcomes A.M for Histology) Fig. 1 shows the CONSORT flowchart for this randomized controlled clinical trial.

### Sample size calculation

According to the previous work by Guarnieri R et al.^15^, minimum total sample size of 36 samples was sufficient to detect the effect size of 0.32 (moderate effect size), with a power (1−β = 0.90) at a significance probability level of *p* ≤ 0.05 to evaluate the ARP technique. According to sample size calculations, there is a 90% chance of correctly rejecting the null hypothesis of no significant effect if each group represented by 12 samples. The sample size was calculated according to G* Power software version 3.1.9.7.

### Inclusion and exclusion criteria

Subjects who required extraction of unrestorable single rooted maxillary anterior or premolar tooth due to endodontic complication, root fracture, trauma, or advanced carious lesions and to be followed by implant placement were enrolled in the study. This area was selected due to its esthetic significane and its frequent need for additional bone grafting procedures either before or during implant placement. Inclusion criteria: (1) Healthy adults; (2) Aged between 20 and 60; (3) Socket defect types in Elian classification I and IIa^16^ (4) Teeth with or without periapical lesions not affecting buccal wall integrity (5) Patients with clinical periodontal health on an intact periodontium and adequate volume that allow for implant placement after follow-up period. Exclusion criteria included: (1) Smokers in the past 5 years, (2) pregnant women, (3) active infections, (4) uncontrolled systemic diseases, and conditions that affect bone and soft tissue healing, (5) history of malignancy or radiotherapy in the past 5 years, (6) history of active bone metabolic disease.

Before extraction, after local anesthesia, the width of the attached gingiva was measured by stretching the lip and subtracting the sulcus or pocket depth from the total width of the attached gingiva- from gingival margin to mucogingival junction-using a Williams graduated periodontal probe^17^. Gingival thickness was measured midbuccally halfway between the mucogingival junction and the free gingival groove in the attached gingiva using an endodontic spreader fitted with a rubber stopper^18^. Using cone beam computed tomography (CBCT), every patient had a preoperative assessment of their alveolar bone dimensions. Before intervention, subjects underwent scaling and root planning with individualized oral hygiene instructions to establish an adequate oral hygiene .

### Surgical protocol

Surgical protocol involved two phases: Phase 1: atraumatic extraction for all groups and ARP interventions for group I and II. Phase two: Core biopsy harvest before implant placement 6 months after extraction for all groups.

### Phase 1: extraction and augmentation

After rinsing their mouths with chlorhexidine mouthwash (Hexitol, ADCO Pharma, Egypt) for one minute, extractions were done atraumatically using periotomes, elevators, and forceps using flapless approach to prevent damage to the buccal cortical plate.The alveolus was thoroughly curetted to remove all the granulation tissue, irrigated with sterile saline and carefully inspected to check the integrity of labial bone. subjects were randomly assigned in group I: There were three layers of materials arranged in a BARP style into the extraction socket: (1) deep collagen layer of collagen sponge (PARASORB^®^ Dental cones, Resorba medical, Germany) filling the socket up to 4–5 mm from the most coronal extensions of the buccal and lingual crest to support the coronal graft; (2) xenograft layer (Bovine bone xenograft, International Biotechnology Company, USA) placed on top of the apical collagen layer to fill the coronal portion of the socket and (3) a superficial coronal collagen sponge layer, used to obtain socket sealing. Group II: sockets were filled up with collagen sponge. Group III (Control): sockets left for spontaneous healing. In all groups, sockets were stabilized by criss cross sutures (absorbable braided polyglycolic acid suture-Taisier-Med, Egypt).

After surgery, postoperative oral analgesics 600 mg of ibuprofen (Kahira Pharm Co., Cairo, Egypt.) every 12 h for 3–4 days and systemic antibiotics Amoxicillin 875 mg and clavulanic acid 125 mg tablets (GlaxoSmith Kline, England) every 12 h for 6 days to avoid postoperative infection. A mouth rinse of 0.12% chlorhexidine, twice daily, was prescribed for plaque control. Sutures were removed after two weeks^19^.

### Phase 2: bone core harvesting and implant placement

A surgical re-entry was performed at the extraction site six months later to collect bone core biopsies and implant placement. After local anesthetic, the grafted region was exposed by reflecting a full-thickness flap. To get the bone core biopsy, a trephine bur measuring 3.0 mm in diameter ( Helmut ZEPF, Germany) was utilized.

### Outcome variables

The primary outcome was alveolar ridge vertical dimensional changes using CBCT. The secondary outcomes were CBCT horizontal ridge dimension changes, soft tissue volume changes and quality of bone formed 6 months after surgery.

### Histological examination

Bone biopsies were taken in formalin 10%, followed by decalcification in 10% formic acid for 2 days, the specimens were then dehydrated through a graded series of isopropyl alcohol concentrations, ranging from 70 to 100%, cleared in xylene, and subsequently embedded in paraffin. Serial sections, each 4 μm thick, were prepared from the paraffin blocks and stained using hematoxylin and eosin stain for histologic and histomorphometric studies. Computerized scanning and analysis of the histoloical slides was performed (Leica Qwin 500, Germany) at Faculty of Dentistry for Girls, Al Azhar University. At least three randomly selected sections of each subject were used to obtain the percentage of total bone (lamellar bone (%) and woven bone (%), residual graft particles (%).

### Radiographic evaluation

CBCT scans at base line and at 6 months were processed using (Planmecca Viso G7 pro) to compare vertical and horizontal bone measurements at before and 6 months after extraction, using the same referencing system that was done individually on each CBCT volume using a free-ware DICOM viewer (Planmeca Romexis viewer 5.3.3.5). Data was acquired using 5.040 scanning time at 100 Kvp and 12.5 mA as exposure with voxel size of 150 Mm and Field of view of 20 cm × 20 cm for all subjects. Romexis 3D imaging software was used to evaluate the ridge defects.

**Fig. 2:**
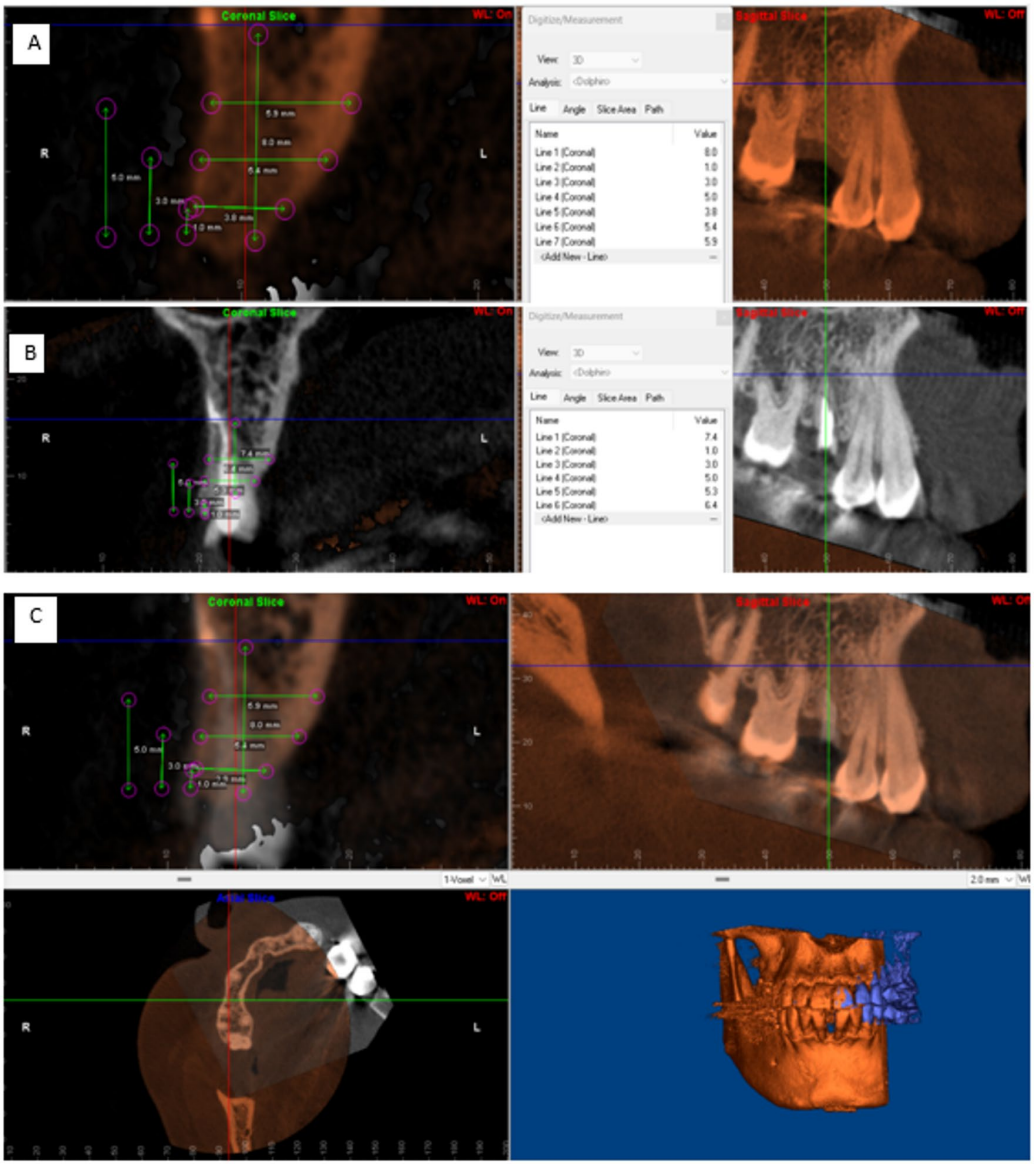
Photographs of bone measurements (height and width) on CBCT cuts showing (**A**); preoperative CBCT for non-restorable upper right second premolar (**B**); measurements after extraction and (**C**) photographs of axial, sagittal, and coronal CBCT cuts showing fusion between primary and secondary images.

After proper postprocessing such as cropping and filtering via “Dolphine imaging version 11.5”, superimposition of the two scans (baseline and 6 months follow up) was done using point based registration method. points were infra-orbital foramen at both sides, anterior and posterior nasal spines, mesiobuccal (MB) cusp tip of each upper first molar if present and in case of any or both are missing the second molar MB cusps were used instead. Subsequently the measurements were made using the same reference points and lines. To set a reference, the most apical point of the socket of tooth planned to be extracted was defined in the base line image. Two reference lines subsequently drawn. The vertical reference line was drawn in the center of the extraction socket starting from the apical reference point. The horizontal reference line was drawn perpendicular to the vertical line at the level of highest alveolar crest. The horizontal reference line was drawn perpendicular to the vertical line at the level of highest alveolar crest. Then the following measurements with respect to these reference points and lines were then performed^20^. The alveolar bone height and the ridge width was measured parallel to the horizontal reference at three different levels 1, 3, and 5 mm from the highest buccal ridge point on the secondary image. Measurements were transferred to excel sheet for calculation and statistical analysis^21^ (Fig. 2A–C).

### Statistical analysis

Statistical analysis for results was performed using one way ANOVA to compare between data followed by Post Hoc test (Tukey) for pairwise comparisons between different groups. Chi-square test of significance was used to compare proportions between two qualitative parameters. The percentage of change was calculated. Intra-rater reliability was evaluated by using intra-class correlation coefficient (ICC) analysis (*p* ≤ 0.05 was considered statistically significant (95% significance level), *p* ≤ 0.001 was considered as highly statistically significant (99% significance level). The Shapiro–Wilk test was employed to guarantee that the data was normal. Statistical evaluation was performed using the SPSS statistical package (version 25, IBM Co. USA).

## Results

The mean age of participants was 37.30 ± 6.65, 36.50 ± 10.64 and 37.50 ± 7.53 years for GP1, GP II and GP III (control) respectively, with no statistically significant difference between groups (P-value = 0.953). A no statistically significant difference was reported between the study groups regarding male/ female distribution(P-value = 0.87). No adverse events have been reported by study subjects. All tested sockets were from upper anterior and premolar areas. And all study groups included socket types I and IIa (Table 1).

**Table 1:** Demographic data of the experimental and control groups.

Demographic data	GPI	GPII	GPIII (control)	*P*-value
Age (years)	37.30 ± 6.65	36.50 ± 10.64	37.50 ± 7.53	0.953
Gender				
Male	3 (25%)	3 (25%)	4 (33.3%)	0.87
Female	9 (75%)	9 (75%)	8 (66.7%)	
Extracted tooth type				
Upper right first premolar	3 (25%)	1 (8.3%)	3 (25%)	0.778
Upper left first premolar	0 (0%)	2 (16.7%)	1 (8.3%)	
Upper right second premolar	2 (16.7%)	3 (25%)	2 (16.7%)	
Upper left second premolar	2 (16.7%)	0 (0%)	2 (16.7%)	
Upper right lateral incisor	1 (8.3%)	0 (0%)	0 (0%)	
Upper left lateral incisor	0 (0%)	1 (8.3%)	0 (0%)	
Upper right central incisor	1 (8.3%)	2 (16.7%)	2 (16.7%)	
Upper left central incisor	1 (8.3%)	1 (8.3%)	0 (0%)	
Upper right canine incisor	0 (0%)	0 (0%)	1 (8.3%)	
Upper left canine incisor	2 (16.7%)	2 (16.7%)	1 (8.3%)	
Socket Type				
Type I	10 (83.3%)	10 (83.3%)	9 (75%)	0.837
Type IIa	2 (16.7%)	2 (16.7%)	3 (25%)	

Data presents as mean ± SD or frequency (%).

### Soft tissue changes

Regarding attached gingiva width at baseline, a no statistically significant difference was observed between the three groups (P-value = 0.963), mean ± SD values were 8.75 ± 1.39, 8.86 ± 0.9 and 8.71 ± 0.49 mm for GP I, GP II and GP III (control) respectively. Six months later, GP III (control) showed the greatest percentage of change (mean ± SD 7 ± 0), while GP II exhibited the lowest values (8.86 ± 0.9), and there was no statistically significant difference between GP I and GP II. A highly significant difference was seen between GP III (control) and the other groups (P-value < 0.001). For attached gingiva thickness, at baseline, a no statistically significant difference was observed between the 3 groups mean ± SD were 1.79 ± 0.39, 1.94 ± 0.18 and 1.86 ± 0.38 for GP III (control), GP I and GP II respectively. After 6 months, although the highest mean values were achieved in GP I (mean ± SD 1.94 ± 0.18) and the lowest one was achieved in GP III (control) (mean ± SD 1.79 ± 0.39), a no statistically significant difference between the three groups was shown (P-value = 0.670) (Table 2). The Intra-rater agreement using the ICC, demonstrated values ranged from 0.879 to 0.998 for width of keratinized gingiva and from 0.869 to 0.996 for thickness of keratinized gingiva, all showing high statistical significance (*p* < 0.001**).

**Table 2:** Clinical measurements of attached gingiva width and thickness and intergroup comparison at baseline and 6 months postoperative.

	Baseline		6 months		Percentage of change		Effect size (95% C.I.)
	Mean ± SD	95% C.I. for mean	Mean ± SD	95% C.I. for mean	Mean ± SD	95% C.I. for mean	
Width of keratinized gingiva							
GP I	8.75 ± 1.39^Aa^	7.36–10.14	8.69 ± 1.39^Aa^	7.3–10.1	0.70 ± 1.97^B^	-0.41–1.81	0.043(-1.116, 1.236)
GP II	8.86 ± 0.9^Aa^	7.96–9.76	8.86 ± 0.9^Aa^	8-9.8	0 ± 0^B^	0–0	3.83(1.333, 2.087)
GP III (Control)	8.71 ± 0.49^Aa^	8.22–9.2	7.0 ± 0.0^Bb^	7 to 7	19.44 ± 4.74^A^	14.7–24.2	3.45(1.292, 2.128)
P-value	0.963		**0.003** ^*^		**< 0.001** ^**^		
Thickness of keratinized gingiva							
GP I	1.94 ± 0.18^**Aa**^	1.84–2.04	1.94 ± 0.18^**Aa**^	1.84–2.04	0.00 ± 0.00	0.00–0.00	0(-0.151, 0.151)
GP II	1.86 ± 0.38^**Aa**^	1.64–2.08	1.86 ± 0.38^**Aa**^	1.64–2.08	0.00 ± 0.00	0.00–0.00	0(-0.321, 0.321)
GP III (Control)	1.79 ± 0.39^**Aa**^	1.57–2.01	1.79 ± 0.39^**Aa**^	1.57–2.01	0.00 ± 0.00	0.00–0.00	0(-0.329, 0.329)
P-value	0.670		0.670		1.000		

Data presents as mean ± SD or frequency (%). Different small letters indicate significant difference at (*p* < 0.05) among means in the same row, Different capital letters indicate significant difference at (*p* < 0.05) among means in the same column. *Significant as P value ≤ 0.05, **highly significant.

### Evaluation of alveolar bone width and height

Assessment of alveolar bone width changes by CBCT was done at different levels 1, 3, 5 mm from the level of crestal bone. For intra-group comparison in GP III (control) and GP II, at 1 mm levels, a highly significant change was observed between pre and post extraction values (*P* ≤ 0.001); at 3 and 5 mm points, a significant difference was shown between pre and post extraction measurements. In GP I, only a significant reduction on pre- and post- extraction bone width was observed at the 1 mm point, whereas these values didn’t show a statistically significant reduction at the 3 and 5 mms points, the overall P-value = 0.05. For inter-group comparison, at baseline, no statistically significant differences were observed between the three groups at 1, 3 and 5 mm level. For the values after 6 months, GP III (control) at 1 mm showed the significantly lowest measurements (mean ± SD) (4 ± 1.28 mm) compared to the other 2 groups (6.06 ± 1.34 and 5.99 ± 0.53 mm) for GP I and GP II respectively with no statistically significant difference shown between them. At the 3 and 5 mms levels, a highly significant difference was shown between the three studied groups (*P* ≤ 0.001), and GP I showed the highest mean width at these reference points compared to the other groups. Regarding alveolar ridge height, for GP III (control), the mean ± SD values were 9.21 ± 1.02 mm before extraction, decreased significantly to 6.91 ± 0.83 mm after 6 months (P-value < 0.001). For GP I, no statistically significant reduction was shown (P-value = 0.477) as mean values were 10.89 ± 2.5 and 9.98 ± 2.5 mm for pre-and post-extraction values respectively. In GP II, no statistically significant change was also reported after extraction in mean height values (P-value = 0.108). As for inter group comparison, the highest change in alveolar ridge height was achieved in GP III (control) and significantly higher than the other 2 groups. GP II showed the least changes with no statistically significant difference between GP I and GP II (Table 3).

**Table 3:** Alveolar ridge width and length CBCT measurements and intra-group comparison between the three measuring points at baseline and 6 months postoperative.

			Baseline		6 months		Percentage of change		Effect size (95% C.I.)
			Mean ± SD	95% C.I. for mean	Mean ± SD	95% C.I. for mean	Mean ± SD	95% C.I. for mean	
Alveolar ridge width	GP I	1 mm	7.33 ± 1.34^**Aa**^	6.57–8.09	6.06 ± 1.34^**Bb**^	5.30–6.82	17.81 ± 3.97^**A**^	15.56–20.06	0.948(0.136, 2.404)
		3 mm	8.08 ± 1.45^**Aa**^	7.26–8.90	7.98 ± 1.42^**Aa**^	7.18–8.78	1.14 ± 2.44^**B**^	-0.24-2.52	0.07(-1.115, 1.315)
		5 mm	8.95 ± 1.46^**Aa**^	8.12–9.78	8.6 ± 1.49^**Aa**^	7.76–9.44	4.06 ± 1.9^**B**^	2.98–5.14	0.237(-0.898, 1.598)
		P-value	0.095		**0.005** ^*^		**< 0.001** ^**^		
	GP II	1 mm	7.39 ± 0.53^**Ba**^	**7.09–7.69**	5.99 ± 0.53^**Bb**^	5.69–6.29	19 ± 2.77^**A**^	17.43–20.57	2.64(0.952, 1.848)
		3 mm	7.94 ± 0.4^**ABa**^	7.71–8.17	6.77 ± 0.5^**ABb**^	6.49–7.05	14.82 ± 2.65^**AB**^	13.32–16.32	2.58(0.787, 1.553)
		5 mm	8.14 ± 0.65^**Aa**^	7.77–8.51	7.01 ± 0.73^**Ab**^	6.60–7.42	13.98 ± 2.85^**B**^	12.37–15.59	1.635(0.545, 1.715)
		P-value	**0.044** ^*^		**0.012** ^*^		**0.007** ^*^		
	GPIII (Control)	1 mm	6.73 ± 0.95^**B**a^	6.19–7.27	4 ± 1.28^**Bb**^	3.28–4.72	41.79 ± 10.3^**A**^	35.96–47.62	2.423(1.776, 3.684)
		3 mm	7.54 ± 1.14^**ABa**^	6.89–8.19	5.39 ± 1.51^**ABb**^	4.54–6.24	29.72 ± 8.85^**B**^	24.71–34.73	1.607(1.018, 3.282)
		5 mm	8.6 ± 1.26^**Aa**^	7.89–9.31	6.94 ± 1.3^**Ab**^	6.20–7.68	19.65 ± 4.05^**C**^	17.36–21.94	1.297(0.577, 2.743)
		P-value	**0.020** ^*^		**0.003** ^*^		**< 0.001** ^**^		
Alveolar ridge length	GP I		10.89 ± 2.5^**Aa**^	9.48–12.30	9.98 ± 2.5^**Aa**^	8.57–11.39	8.68 ± 2.69^**B**^	7.16–10.20	0.364(-1.208, 3.028)
	GP II		10.8 ± 1.03^**Aa**^	10.22–11.38	9.87 ± 0.97^**Aa**^	9.32–10.42	8.61 ± 2.14^**B**^	7.40–9.82	0.93(0.084, 1.776)
	GP III (Control)		9.21 ± 1.02^**Aa**^	8.63–9.79	6.91 ± 0.83^**Bb**^	6.44–7.38	24.99 ± 2.01^**A**^	23.85–26.13	2.47(1.512, 3.088)
	P-value		0.144		**0.004** ^*^		**< 0.001** ^**^		

Data presents as mean ± SD or frequency (%). Different small letters indicate significant difference at (*p* < 0.05) among means in the same row -Different capital letters indicate significant difference at (*p* < 0.05) among means in the same column. *Significant as P value ≤ 0.05, **highly significant.

### Histological assessment

**Fig. 3:**
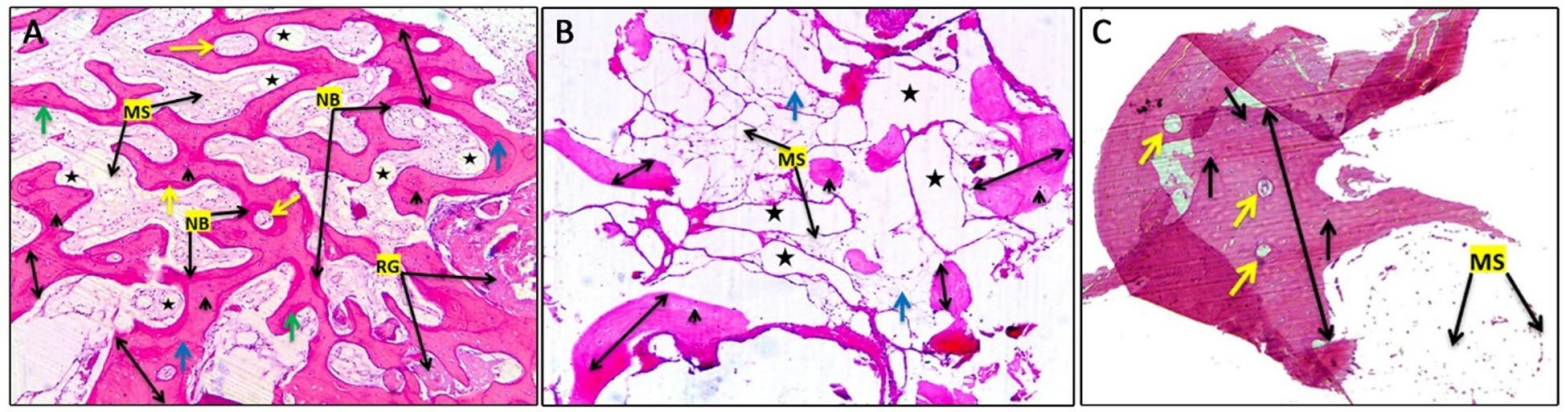
(**A**) Histological analysis of GP I Orig. Mag. × 40 NB: new bone, RG: residual graft, MS: marrow spaces, bone trabeculae thickness (double head arrows), Haversian canal (yellow arrows), reversal line (blue arrows), resting line (green arrows), osteocytes (arrow head), dilated blood vessels (stars), (**B**) Histological analysis of GPII, Orig. Mag. × 40 Scattered small bone spicules (double head arrows), MS: fatty marrow spaces, fat cells (blue arrows), osteocytes (arrow head), and edematous area (stars), (**C**) Histological analysis of GP III (control), Orig. Mag. × 40. This part of socket revealed large piece of bone spicules (double head arrows), osteocytes (arrows), Haversian canal (yellow arrows), MS: marrow spaces.

For all groups, microscopic image of H& E-stained sections showed that the specimens were almost filled by large and interconnected bone trabeculae organized in radiated plates like structure. Many entrapped osteocytes within the trabeculae and multiple osteoblasts were seen at the periphery. In some areas of the new bone, many of reversal and resting lines oriented in different directions and approximate to each other’s, while others show Haversian canal formation. The marrow spaces between those bone trabeculae were filled with dilated blood vessels as well as adipocytes. While some areas exhibited residual graft in GP I and II. In GP II and III (control), granulation tissue with some inflammatory cells were seen (Fig. 3A–C).

### Histomorphometrical analysis

The highest mean value of area% of lamellar bone was recorded in GP I: (54.80 ± 6.69), followed by GP II (27.34 ± 5.74) and then GP III (control) (22.21 ± 3.66), with no statistically significant difference between GP II and III (control). The highest mean value of area% of woven bone was shown in GP II: (27.71 ± 4.85), followed by GP III (control) (21.38 ± 3.43) and then GP I: (14.69 ± 3.57). Tukey’s post hoc test revealed multiple significant difference between groups. Regarding the residual graft particles, the highest mean value of area% of residual graft was recorded in GP I (21.32 ± 1.86), followed by GP II (4.19 ± 1.40), with a statistically significant difference between groups (*P* = 0.001) (Table 4).

**Table 4:** Histomorphometric data of area % of lamellar bone, woven bone and the amount of residual graft in groups.

		Mean	±SD	±SE	95% C.I. for mean		Min.	Max.	p-value
					Lower	Upper			
Area % of lamellar bone	GP I	54.80^A^	6.69	2.36	49.21	60.39	40.96	60.75	0.001*
	GP II	27.34^B^	5.74	2.03	22.55	32.14	16.82	34.89	
	GP III (control)	22.21^B^	3.66	1.29	19.14	25.27	18.47	29.81	
Area % of woven bone	GP I	14.69^C^	3.57	1.26	11.70	17.68	8.96	17.04	0.001**
	GP II	27.71^A^	4.85	1.72	23.65	31.76	19.89	32.55	
	GP III (Control)	21.38^B^	3.43	1.21	18.51	24.25	17.56	25.35	
Area % of the amount of residual graft	GP I	21.32	1.86	0.66	19.77	22.87	18.97	23.56	0.001*
	GP II	4.19	1.40	0.49	3.02	5.36	3.15	7.37	

Data presents as mean ± SD or frequency (%). *Significant as P value ≤ 0.05; **highly significant p value ≤ 0.001. Different capital letters indicate significant difference at (*p* < 0.05) among means in the same column. Different small letters indicate significant difference at (*p* < 0.05) among means in the same row.

## Discussion

To counteract physiological bone loss that happens after tooth extraction, ARP techniques using bone graft and additional absorbable membrane have been widely utilized. Although socket grafting cannot prevent the resorption of facial and palatal bone wall, it helps to preserve the bone volume, leading to better clinical outcomes^22^. Xenograft is a feasible bone graft, being porous, resorbable, osteoconductive, biocompatible and available in unlimited quantities with no donor site harmful effect^23^. This work compared for the first time between Biologically oriented alveolar ridge preservation (BARP) technique versus collagen sponge alone in ARP. Both treatments showed favorable results regarding alveolar ridge width and height reduction and soft tissues dimensional changes compared to negative controls extraction sockets. The two intervention groups caused a significant maintenance of gingival width compared to controls. But when it came to the thickness of the keratinized gingiva, none of the three groups differed significantly. According to Pramstraller et al., the superficial layer of collagen sponge at socket entrance, causes clot and graft stabilization, allowing for successful re-epithelialization, leading to minimal changes in the apico-coronal dimension of the keratinized peri-implant mucosa^12^. The shrinkage of the soft tissues outlining the cervico-buccal region is known to be attributed to the influence of buccal bone remodeling of the extraction socket and this cannot be completely avoided by socket grafting. Further, the tooth offered transgingival support prior to extraction which is another important factor^24^.

Although the collagen sponge was exposed in oral cavity after flap suturing in intervention groups, a new soft tissue, was formed without signs of mucosal inflammation or immunological reaction in the follow-up period, in accordance with earlier findings^11,12^. It was reported that the primary closure of surgical flaps did not show beneficial effects on preserving the alveolar bone in case of ridge preservation technique^25,26^.

In ARP procedures based on socket grafting, the main bulk of volume reduction occurs in the coronal third (up to 30%), while of no to limited effect in the remaining apical two thirds. In grafted alveolus, residual graft particles embedded into the newly formed bone are often present, delaying the rate of bone deposition and mineralization. Thus, the need to extend bone grafting beyond coronal third of the socket seems questionable^10^. In accordance, our results showed remnants of residual graft particles embedded into the newly formed bone in bone specimens of GP I. Moreover, there was an overall bone width change with the largest percent of change at 1 mm and the smallest percent of change in 3 mm and 5 mm, similarly, for GP II, there was an overall reduction in the bone width with the largest percent of change coronally at 1 mm and the smallest percent of change at 3 and 5 mm levels. As for the GP III (control), the overall volume reduction at the three measuring points was significantly higher than the other two groups. Our results are in agreement with previous findings^12,27^ .

The effectiveness of collagen sponge as a biocompatible material has been shown in ARP through wound protection, clot stabilization, and granulation tissue formation causing uncomplicated wound healing. Further, collagen exhibits a slow degradation as well as a preferable environment for osteoblastic adhesion and differentiation^28^. In this work, collagen sponge served for both socket preservation and socket seal in GP II and in accordance with previous data, no clinical adverse reaction or post extraction complications were observed^28,29^. Our results showed that collagen sponge in GP II resulted in significant preservation in post extraction soft and hard tissues changes compared to naturally healed extraction socket, in agreement with previous data^11,29,30^. Further, this work showed that collagen sponge alone was as equally effective as BARP technique regarding soft and hard tissue outcomes. As a socket seal material, it was recently reported that collagen sponge was as effective as free gingival graft in preserving bone dimensions after extraction^31^. Results of GP I and II in our work could be explained by what was earlier reported by Pramstraller M et al.^12^; as the deep collagen layer in GP I and II seems to efficiently support the clot during the bone healing process, moreover, this layer in GP I was able to maintain the xenograft particles in the coronal portion of the socket during the tissue maturation period. However, we can propose that collagen sponge alone for socket grafting, can do the same function of xenografts in the coronal third of GP II causing maintenance of tissue dimensions during healing.

One of this work strengths is the histologic and histormorphometric evaluation, with extended 6 months follow up period, unlike others with 3 or 4 months follow up^28,32^. It was earlier reported that longer healing time (18–20 weeks) in ARP studies with different grafts allowed for increased amount of vital bone formation compared to the short follow up studies^33,34^. New bone formation was evident in all studied groups. Increased lamellar bone in the healing socket plays a key role in achieving successful osseointegration and long-term stability of the implant^35^.The mean area percent of lamellar bone and residual graft particles at sites grafted with bovine xenograft in GP I were 54.81% and 21.32%, respectively, which is consistent with earlier findings^1,28^, further, area % of lamellar bone was significantly higher in GP I compared to the rest of the groups, and GP III (control) showed the lowest area % of lamellar bone with no statistically significant difference between GP II and III (control).

No inflammatory reaction was reported in specimens of GP I – in agreement with Bureekanchan et al.^1^.

however, in GP II and III (control) specimens, sporadic areas of granulation tissue formation and inflammatory cells were seen, disagreeing with Kairy et al.^28^ who reported absence of inflammation in ARP using collagen sponge alone. This could be attributed to the different types and brands of collagen sponges used in both studies. Another possible cause might be an allergic reaction to collagen, while collagen is considered a weak antigen, it can cause allergic reactions in rare cases. Clinical assessments indicated that 2–4% of the general population exhibit an allergic reaction to bovine type I collagen^11,36,37^.Further, the absence of inflammatory cells in group I might be due to the layering technique of BARP that creates environment that supports sequential healing and enhance the blood supply to accelerate collagen resorption, reducing inflammation and encourage bone regeneration, especially that the amount of collagen used in group I was much less than that used in group II. Another finding in GP II is the reported average area percent of the residual graft (4.19%), unlike previous studies who reported complete resorption of collagen sponge with no graft residuals in collagen grafted sites^28,38^. These controversial outcomes could be related to the difference in evaluation method and the follow up period. To overcome the issue of residual graft materials, a recently introduced engineered alternative to xenografts with tunable properties relevant to ARP like ideal biocompatibility and high osteoconductivity might be useful^39^.

## Conclusions

It can be concluded that although both tested groups demonstrated favorable clinical and radiographic outcomes in terms of limiting soft tissues and bone dimensional alteration of the alveolar ridge after teeth extraction, however, collagen sponge and xenografts group exhibited histological indication of greater percentage of lamellar bone formation and less inflammatory reaction. This work highlights the relevance of histologic examinations in ARP studies.

## Data Availability

Data available on request duo to privacy/ethical restrictions.
